# “*A heart of the man is lighter than that of the woman…”* exploring men’s motivation and capability to access HIV services in Lusaka, Zambia: findings from the Yaba Guy Che study

**DOI:** 10.1186/s12889-025-23703-2

**Published:** 2025-10-06

**Authors:** Chisanga Mwansa, Mwelwa M. Phiri, Steve Belemu, Loyd Kalekanya, Charles Banda, Lucheka Sigande, Helen Ayles, Musonda Simwinga, Bernadette Hensen

**Affiliations:** 1 Zambart, Unza Ridgeway Campus, Off Nationalist Road, Lusaka, P.O Box 50697, Zambia; 2 Department of Clinical Research, Faculty of Infectious and Tropical Diseases, London School of Hygiene and Tropical Medicine, London, UK; 3 Department of Public Health, Institute of Tropical Medicine, Antwerp, Belgium

**Keywords:** Zambia, HIV testing, HIV services, Stigma, Men, Norms, Socio-ecological framework

## Abstract

**Background:**

In southern Africa, men are less likely than women to access HIV services, including HIV testing, antiretroviral therapy (ART), and HIV prevention services. As a result, men living with HIV are less likely to be virally suppressed and more likely to transmit HIV than their female peers. Using the socio-ecological model as a framework, we explored factors that influence men’s motivation and capability to access available HIV services, including how social norms and social networks influence men’s engagement with services, in Lusaka, Zambia.

**Methods:**

We conducted seven focus group discussions (FGDs) with men and women in an urban community in Lusaka. Five FGDs were conducted with men; two with young men aged 20–24; two with men aged 25–35 and one with men aged 20–35. We conducted two FGDs with women, stratified by age 18–24 and 25–35. The total number of participants was 70. Data were coded and analysed thematically.

**Results:**

Pervasive negative community narratives around HIV, negative social and gender norms, the influence of men’s social networks, including stigma related to a positive HIV test result and fear of social isolation, were among the key factors influencing men’s access to HIV services. For HIV testing, the organization and delivery of services in health facilities, including location of HIV testing, waiting times, and likelihood of being seen accessing services, dissuaded men from testing for HIV. In general, health facilities were seen as women’s spaces and unresponsive to men’s needs. However, provider-initiated initiatives, including couples testing in antenatal care and an offer of HIV testing prior to medical male circumcision, and community-based HIV testing facilitated service use. Though condoms were the primary HIV prevention tool mentioned by study participants, norms of their use in marriage and sexual relations limited use.

**Conclusions:**

Despite HIV having evolved to a chronic condition and various HIV prevention tools available, fear, social isolation, stigma, and harmful gender norms continue to negatively impact men’s motivation and capability to engage with available HIV services. Measures to facilitate men’s use of these services should consider how to increase social support alongside the delivery of services in spaces that meet men’s needs.

## Background

Globally, over 50% of people living with HIV reside in the Eastern and Southern African regions, making these regions the most affected by the HIV epidemic [1]. In Southern Africa, men are less likely than women to access HIV services, including HIV testing, antiretroviral therapy (ART), and HIV prevention services [2–6]. As a consequence, fewer men compared to women know their HIV status or they get diagnosed with HIV at later stages of infection and have lower ART coverage and adherence [6, 7]. In 2017, UNAIDS estimated that men in sub-Saharan Africa were 20% less likely to test for HIV and, men living with HIV, 27% less likely to initiate HIV treatment compared to women [8]. At this time, men’s lower uptake of HIV-related services was coined the “blind spot” in the HIV response, and addressing this disparity defined as key to achieving HIV elimination by 2030 [6, 9].

Studies in Zambia have shown gaps in men’s access to HIV services and the need to tailor services to men [10, 11]. According to the 2018 Zambia Demographic and Health Survey, 52% of men aged 15 to 59 had tested for HIV and received the result of this test in the last 12 months compared to 64% of women aged 15 to 49 [12]. Analysis of phylogenetic data from the HPTN-071 universal HIV testing-and-treat trial found that, despite their lower incidence and prevalence of HIV, men aged 25 to 40 years were two-times more likely than women of the same age to transmit HIV [13]. Men’s lower engagement in HIV testing, prevention and care not only has consequences for their own health but also that of their sexual partners [14, 15].

Men’s limited access to healthcare services is affected by a myriad of factors at individual-level through to community-level; for example, norms related to masculinity often emphasize the importance of being strong and in control and are therefore at odds with the “patient” persona [16–20]. Additionally, a number of individual, cultural, and societal factors, including the opportunity costs associated with accessing healthcare, continue to undermine men’s access to HIV services [21]. The organization and structure of the public health system also influence men’s motivation to access services, with the system traditionally being less responsive to men’s needs and preferences. Most HIV services have targeted women, placing particular emphasis on maternal and child health [22]. Although, more recently, there have been male circumcision and HIV testing campaigns targeting men [23, 24], and couples HIV testing and prevention services [25], there remains less focus on men’s health.

A goal of the 2022–2026 Zambian National Health Strategic Plan is to reduce HIV incidence from 28,000 to 15,000 by 2026 [26]. Meeting national and global HIV elimination targets requires concerted efforts to reach men with HIV testing services and support their linkage to prevention and treatment services. Adapting health systems to the needs of men requires an in-depth understanding of the factors that influence men’s motivation and capability to engage with and utilize HIV services in the context of increased availability of HIV testing, care and prevention services [27–29]. Using the socio-ecological framework [24, 25], which acknowledges that a complex interplay between individual through to societal factors influence individual behaviour, we conducted a qualitative study in Lusaka, Zambia, to explore the factors that influence men’s motivation and capability to access and use HIV services, with a focus on how social networks and norms influence men’s engagement with services.

## Methods

### Study location and population

This qualitative study was conducted between April and December 2022 in a high-density urban community in Lusaka, Zambia. This study was part of a larger formative research study whose overall aim was to use a community and health systems-thinking approach to co-develop an intervention to increase men’s uptake of HIV-related services, including HIV testing services. As part of this study, we conducted participatory qualitative research with men aged 20–35 years and women aged 18–35 years, resident in the community. The age limit for men was selected based on the above-mentioned evidence that men of this age group are two times more likely to transmit HIV; for women, we chose a lower age limit as men are likely to have younger partners [30, 31].

The participants were purposively selected based on sex, age, and residence in the study community. Age distribution was considered at recruitment through a checklist. Participants were eligible if they were between the ages of 20–35 (male) and 18–35 (female) and their primary residence was in the study community for at least one year. Recruitment was done by research assistants (LK and CB) familiar with the community through active outreach in public spaces such as markets, football grounds and known community hotspots for men, and through word-of-mouth referrals from local networks. During informal conversations, interested individuals were approached, and screening was then conducted using an eligibility checklist based on residency and age. This purposive strategy aimed to ensure variation across age groups and geographic zones within the community. We conducted 7 focus group discussions (FGDs) with a total of 50 males and 20 females.

Table 1 summarizes the age and sex breakdown of participants across the FGDs.

**Table 1:** Socio-demographic characteristics of the study participants

Sex and Age	Age Range	Male (no.)	Female (no.)	Total per Discussion
Men 20–24	20–23 years	10	-	10
Men 20–24	20–24 years	08	-	08
Men 25–35	26–35 years	12	-	12
Men 25–35	30–35 years	10	-	10
Men 20–35	21–33 years	10	-	10
Women 18–24	18–22 years	-	10	10
Women 25–35	25–34 years	-	10	10
Total		**50**	**20**	**70**

### Theoretical framework

**Fig. 1:**
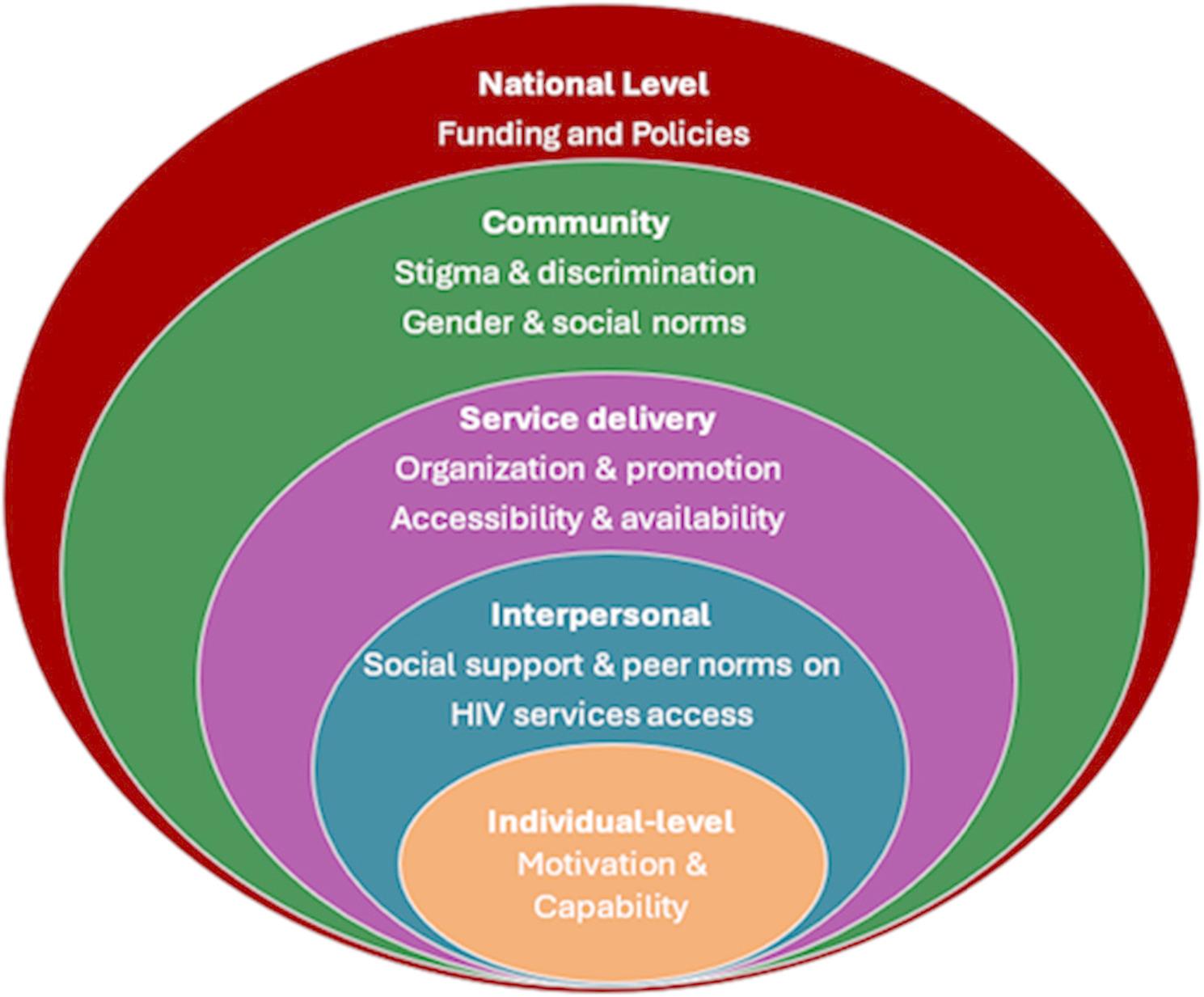
The socio-ecological model

The socio-ecological model [32, 33] was used as a framework for conceptualizing the interacting factors that influence men’s access to HIV-related services. The socio-ecological model suggests that individual behaviour is influenced by policies, institutions and systems, the community, peers, and family. The model can be useful in showing how these interconnected levels of influence affect health outcomes for men. It accounts for individual-level factors like attitudes and knowledge while also recognising interpersonal dynamics like stigma, community-level influences like cultural norms, and structural barriers like health policies and accessibility issues. Addressing the individual-level factors and broader determinants of men’s access to HIV services helps in designing responsive and sustainable interventions for men (see figure 1).

### Data collection

The FGDs aimed to define the HIV services available and accessible to men and to understand factors influencing access to these available services, including how social networks and social norms influence access. We developed discussion tools that combined a social network mapping activity [33, 34], community mapping, and a discussion of factors at different levels of the socio-ecological model that affected men’s motivation and their perceived capability to access and use HIV services.

A pilot discussion was conducted to ensure the FGD tools were clear, understandable, and applicable to the participants. Thereafter, the tools were adjusted accordingly. In adapting the FGD tool, the social network mapping transitioned from visual diagrams with concentric circles to placards representing key social actors (e.g., individuals, families, peers, clinic settings, and community visuals). Discussions then revolved around social norms, reference groups, and valued opinions across the different levels of the socio-ecological model, highlighting how social support, approval, disapproval, and information flow shaped behaviours.

To ensure the study captured different perceptions and experiences from men, FGDs were stratified by age: 20–24 years and 25–35 years. We also had a combined group of men 20–35 years. Additionally, we conducted two discussions with women 18–24 years and 25–35 years old to capture their views and perceptions on the drivers of men’s motivation and capability to access HIV services. All FGDs were conducted by trained researchers and facilitators (CM and SB). To facilitate ease of communication and allow participants to express themselves freely, the discussions were conducted in Nyanja, English, or Bemba, depending on participants’ language preference. Participant recruitment was conducted by research assistants LK and CB, who are familiar with the study community. FGDs were held in a conveniently located venue in the community which was easily accessible to participants and one they felt safe and comfortable to be in. Refreshments and a meal or snacks were provided as compensation.

### Social network mapping

The FGDs included a social network mapping that aimed to understand men’s reference groups [32] and flow of HIV information in their social networks. These reference groups are the people whose opinions about HIV matter the most to men when it comes to accessing HIV services. By identifying these groups, it becomes possible to target them programmatically to advance social and behaviour change because of their influential position in society. The exercise also sought to understand the communication flow within men’s social networks by identifying the individuals they talk to about HIV and HIV services access. This information helps to assess how communication about HIV is being hindered or furthered within men’s social networks and to identify areas that can be improved. Therefore, we asked men who in their social network they talked to about HIV and whose opinions they cared about as well as who is likely to influence their access to HIV services. We also asked them if they thought other men in the community were accessing HIV services and whether or not they would access HIV services if they thought a significant number of people in the community were.

### Data analysis

Thematic analysis was used to analyse the data. The objectives and socio-ecological framework guided the analysis of findings. All FGDs were audio recorded and transcribed verbatim in English. Thereafter, all transcripts were cleaned and checked against the audio for accuracy. Researchers went through the raw data several times to familiarize themselves with the content before assigning preliminary codes. Thereafter a codebook in line with the socio-ecological framework was developed, and deductive codes were defined. This initial coding led to the development of themes across key topics. Emerging themes were further identified inductively by conducting intra-group analysis by age and sex. ATLAS.ti qualitative analysis software was used to code the data. The analysis was led by CM in collaboration with three researchers, ensuring triangulation and validation of themes.

### Ethical approval

The study was conducted in accordance with the tenets of the Declaration of Helsinki. Written informed consent was obtained from all the participants before they participated in the discussions. The London School of Hygiene and Tropical Medicine (Ref: 26713) and the University of Zambia Biomedical Research Ethics Committee (Ref: 2374-2021) approved the study. The study received regulatory approval from the Zambia National Health Research Authority (NHRA).

## Results

The FGDs, including the social network mapping, revealed interacting factors that influence men’s access to HIV services, from community-level perceptions and social norms related to men’s uptake of HIV services to limited knowledge of HIV prevention methods among men, and the limited responsiveness of the health system to men’s needs and preferences. Further, results showed how men’s social networks play a dual role in influencing their access to HIV services by providing critical support and encouragement on one hand while reinforcing stigma and propagating harmful myths on the other.

### Community-level influences on men’s access to HIV services

In this section, we highlight how normative behaviours, stigma, and information at community level intersect to influence men’s access to HIV services.

At community level, limited access to accurate information on HIV services entrenched negative narratives and reduced men’s capability to engage with available services. Misinformation about HIV and on HIV as a fatal diagnosis rather than a manageable chronic condition, and community stigma continue to shape men’s motivation to access HIV services:

*“I think the issue is with sensitization because a lot of people think that once you test positive for HIV then that will be the end, you will die and you will lose weight and they start looking like a mosquito [chuckles]”_* *Man_20–-24 years*

*“They give each other wrong information… there is a rumor that came out that if you go to the clinic then you will be exposed and people will know about your status so that is what they keep sharing amongst themselves and they believe that to be true”_ *Young woman_25-35 years.

When probed, men showed a relative lack of knowledge of HIV prevention methods, such as pre-exposure prophylaxis (PrEP) and post-exposure prophylaxis (PEP), treatment as prevention, and did refer to voluntary medical male circumcision (VMMC)primarily as a hygiene measure rather than a prevention strategy, viewing it less as a way to reduce HIV risk and more as a social expectation. Some men in this research saw circumcision as something they ought to do for women rather than for their own health benefits, often influenced by external pressure:

*“They [parents] used to say that, ‘it is your problem, you are growing up; your wife will be refusing’ [chuckles]”_ Young Man_20–24 years.*

*“Back in 2015 I was going after this girl and she was refusing to have sex with me before testing for HIV. She also demanded that I should be circumcised and I was wondering what she was up to; I gave her a lot of excuses and I said that I was busy with work”_*Man_25–35 years.

In contrast, men talked extensively about condoms as an HIV prevention method and the challenges with accessing and utilising them. They also acknowledged the need for accessing HIV information from credible sources like the health facility. This lack of knowledge about HIV prevention and treatment contributed, in part, to amplifying negative myths around HIV. They also perpetuated narratives, like imminent death, if one is found positive.

### Normative beliefs

FGD participants were asked if they thought men in the community were accessing HIV testing services to ascertain perceived descriptive norms of HIV testing among men. Descriptive norms within the socio-ecological model refer to typical behaviour within a given social context or what is typically done. They are said to influence behaviour based on what people think is widely acceptable or practised, in this case, HIV testing among men. Overwhelmingly, study participants mentioned that they did not think men were testing for HIV and that, in comparison to women, very few men were testing for HIV. However, when asked if they would be motivated to access HIV testing services if they knew most men in the community were testing for HIV, the majority of men responded affirmatively:

*“Yes…because it would help those who do not have the courage to get up and go for testing. If I may give an example, say they announced that in January the percentage of men who know their HIV status is say, 20%, and by the time it is February they say that the percentage has increased to 30% or 40%, automatically, I would also want to be among the people making the percentage for the coming month, it may encourage me to test for HIV”* _Man_25–35 years.

Gender norms affected men’s motivation to access available services. Cultural masculinity narratives that equate health-seeking behaviour with weakness, deterred men from seeking help:

*“What I've noticed about men is that they are very scared of diseases. That is the one thing they are so scared of; the thought of being positive is what makes it difficult for men and that is why when a man goes to the hospital then he is indeed in pain…they are just scared… they are not willing to test. They don't even say anything” _ Women*_25–35 years.

When asked why women were more likely to access HIV services, the response was that women have more opportunities to be at the clinic. Women are the primary caregivers therefore, if a child falls ill, the mother is expected to take the child to the clinic. This also intersects with how clinics are organized with a heavier focus on mother and child health services. Additionally, men and women held the belief that women were more adept at handling diseases and illnesses, including an HIV diagnosis. Women also held the notion that women are more courageous and able to deal with the psychological burden of living with HIV, hence the disparity in access to HIV services:

“*A heart of the man is lighter than that of the woman…a woman is able to hold her heart [remain calm], for example, if they tell her to say, your status is like this, she can handle herself better than a man…for a man, even when they have stomach pains they become weaker than the women…women have that strength to hold themselves better in such situations and that is why a lot of men are scared to go and test for HIV”*_Woman_ 25–35 years.

These perceptions of male emotional vulnerability affected their willingness to seek HIV care.

### Health systems-related factors

The organisation and delivery of HIV services had a strong influence on men’smotivation and capability to access HIV services. The majority of men who had accessed HIV testing reported accessing it through provider-initiated HIV testing. Similarly, community HIV testing initiatives were said to facilitate access and utilization of services as they offered convenience and privacy. Ease of access, proximity, and ability to access services without being seen by others and the age and sex of the service provider were all seen as factors motivating access to HIV services.

On the other hand, the location of HIV services within health facilities, mandatory HIV testing, the availability of family planning clinics, and other services primarily focused on maternal and child health, as well as the alleged lack of male-targeted HIV sensitization, discouraged men from testing.

For ART, the location of ART departments within health facilities was a major barrier for men. Participants discussed how the majority of community members know the layout of the facilities, including what services are offered in which departments, hence standing in a queue for HIV testing at the ART department, for example, created a ‘risk of being seen’ and potentially labelled as sick and on treatment:

*“When we go to this clinic there are so many familiar faces and if you turn going that way [ART dept.]then everyone knows that you’re going to test for HIV; so if I am a resident here, it is better I go and test from S.O.S or Zingalume or Chawama where no one knows me, and I am free” _*Man_ 20–35 years.

*“…When accessing drugs [ARVs], people who know you might see you on the queue…that is why some people would rather go to XXX hospital [outside the community] due to fear of being seen… they would rather go to private clinics in town” _ Young Women_ 18–24 years.*

Policies like Opt-Out provider-initiated HIV testing were widely understood as mandatory HIV testing policy for all who visited the government health facilities, and this created fear among men and reluctance to access the health facility for services in general. The alleged lack of male-targeted HIV sensitization or entry points to HIV services contributed to the perception of health facilities as female spaces, and limited men’s capability to access services. Linking prevention tools to mandatory testing also restricted access to these services. Overall, structural inefficiencies such as visible and dedicated ART clinics and issues related to long waiting hours at the clinic, worsened perception of these barriers:

*“When you arrive at the clinic you have to be in a queue. In that queue, there will be people who have malaria, pregnant women, and so on… there you are and you only want to do an HIV test but you have to queue up… so no wonder we do not like going there.”* _Men 20–24 years.

Negative experiences with healthcare providers while accessing services create a barrier to consistent engagement with the services. The reported non-professional attitudes of some service providers, who were considered rude to patients, were discouraging for some men who valued respectful and professional interactions with the providers:

*“Sometimes you are willing to test for HIV but the people at the clinic will not give you the encouragement needed instead they will start talking to you as if it is your fault somehow, but it is not your fault, and you did not want to be in that situation” _ *Man_20–24 years.

Some men, and women, hence preferred to have gender-matched service provision where male providers serve men and female providers for women to enhance comfort and trust. Others prioritized age and experience over gender:

*“I do not agree with what they're saying that a man should be the one testing because it means that women will be disadvantaged…. As men we can still access services from older women; there are older women there [clinic]… It is ideal to find somebody with experience not whereby the person who is testing you is very young” _* Man_ 20–35 years.

### Inter-personal level factors influencing access to HIV services

Social support and influence from individuals from within men’s social networks, including family members, and female sexual partners’ ability to influence men to access HIV testing services, motivated men to access these services. Partners’ role in motivating men to access HIV services was crucial despite being mostly framed as relational rather than health-oriented.

Women were said to influence men by inviting their spouses to attend couples HIV testing at the health facility or a sexual partner asking men to HIV test, with some men reporting that they feared losing their partner (referred to as “property” by some participants) if they did not oblige:

“*A female can ask you to go and test for HIV and if you refuse, she can just tell you one word to say then you and I are over…then you start thinking to yourself should I lose this property, no… you will just accept to say yes, let us go and test” _ *Man_25–35 years.

While female partners played a pivotal role in encouraging men to test for HIV, participants expressed a tension between relying on their partners and a fear of abandonment or blame should they test positive. This could be contrasted with the lack of male-to-male support, where most men feared talking about HIV or letting their friends and peers know about their HIV status for fear of “being exposed”. On the other hand, men's motivation to seek HIV testing services sometimes came from other members of their household or from living with someone who was known to be living with HIV. For younger men, this influence came from their parents and siblings in the form of a gentle nudge to access HIV testing services whenever they had an illness:

*“People from the household can talk about it, maybe when you're complaining about body pains or a headache then someone will pass a comment saying that you should even be going to the clinic to test for HIV, maybe you are infected.” *_Man *_*20–24 years*.*

Women emphasized their role in making their partners seek HIV services by sometimes using the relationship as a negotiating tool:

*“It is easy for them married women to ask their husbands to test because he knows if he refuses they will be having fights it could mean he is having sexual affairs somewhere.”_*Women_18–24 Years.

Although a spouse or partner could motivate HIV testing, men also avoided testing for fear of abandonment by their spouse or girlfriend, or to avoid confrontation or blame from their partners. Norms and traditions inhibiting discussions around sex in the family impacted men’s motivation to engage with HIV services.

### Barriers within male peer networks

Fear of inadvertent disclosure of an HIV-positive status among friends and subsequent weaponization of this information to isolate them also discouraged men from accessing HIV testing:

*“The issue of discrimination is scary because if I go with someone, automatically when I come out with the results slip, my friend would want to know the contents of that slip; is it positive or is it negative?... I will just notice that when I walk around in the community, people will be pointing fingers at me because he has told everyone my business.”*_Man_25–35 years.

Because of this fear, men were skeptical about discussing or accessing HIV testing with other men and peers in their social network; indicating that friends could negatively impact HIV testing behaviors. Fear of stigma and gossip among family and peers was a recurring theme that prevented men from accessing HIV services. Some men feared that they would be perceived by their friends to be showing off their knowledge of HIV if they suddenly suggested that their friends test for HIV. They also feared that if they tested with their friends, those friends would find out about their HIV status and expose them:

*“You cannot tell your friends because these friends will even scorn you when you lose a little bit of weight, and they even start saying this guy is not okay and so on now what more a situation where you are HIV positive, and you even share with them. I can never do that; I can never tell my friends about my status” *_Man_20–24 years.

This lack of trust among men and peers in their social networks as well as their inability to discuss issues around HIV or seek HIV-related services created an information gap. This led to the sharing of inaccurate HIV information and further normalized men's limited access to and use of HIV-related services. For example, some men still believed that they could determine a woman’s HIV status based on her looks and therefore, avoid using condoms and HIV testing services:

“*OK, it just depends on you as an individual and the kind of women you have sex with. If they look a certain type of way, yes you would be scared…It just depends on the kind of women that you have sex with…if you have two or three girlfriends, you know them but if a guy is just hooking up with women from the bars yes, one would be scared to test” _ *Man_20–24 years.

Social networks, beyond sexual partners, also played a dual role in influencing men’s access to HIV services. They provide crucial social support through trusted friendships but also propagated stigma and misinformation. Social network mapping highlighted these relational dynamics and added depth to the interpersonal and community levels of the socio-ecological model also exposing how community norms and misinformation are perpetuated. This highlighted the need for targeted educational and stigma reduction campaigns.

### Individual-level factors affecting access to HIV-related services

Individual-level motivators for accessing HIV services included deterioration of physical health and self-perceived HIV risk, including suspected partner infidelity. Poor health or physical frailty were reported to be a key motivator for men to access HIV-related services, particularly HIV testing. However, most men would only consider testing for HIV if they were physically in pain:

*“The only time you see men testing at the clinic is when they are very sick, and they are in a coma and their relatives just pick them and take them…but men never voluntarily visit the health facility to test; it is very difficult for men”* _Man_25–35 years.

Barriers at individual level were sometimes driven by a misjudged self-assesment which in turn fostered complacency for seeking HIV services. Participants frequently rationalized their reluctance to undergo HIV testing by associating it with a sense of masculine toughness, linking physical wellness with invulnerability. Additionally, their hesitation was intensified by the anxiety that receiving a positive diagnosis might diminish their perceived social standing and strength:

*“For example, if you know that you are not moving in the right way [sexually active], automatically you know that if I go there, I will be found to be HIV positive…that will give you the fear because you know that when you go there you will be found to be HIV positive” _ *Man_25–35 years.

Another recurring theme was that most men use a lack of sex or extended periods of abstinence as a yardstick for risk of infection. Sometimes men perceived themselves as not at risk based on good self-perceived physical health after condomless sex, as explained by a participant below:

*“…you will start having thoughts like if I have sex with this person without a condom what will happen? But if you have sex with her and you notice that maybe one or two years have gone by and nothing happens, then you can judge to say maybe this person is just fine.”* _Man_20–24 years.

Peer norms often discourage health-seeking behaviour while promoting risky practices. Men also showed a certain propensity to take risks and make decisions about their sexual health, which they knew were likely not in their favour, but these decisions were also rooted in negative gender norms and masculinity. For example, men were still having condomless sex despite knowing the potential consequence of HIV infection. A young man explained that for men, the only sex that counts as sex is condomless sex, as it “shows that you are the owner.”

 “*Going to the clinic trying to access free condoms as if the girl will be waiting for you or as if that is your ‘property’? She can even escape [chuckles]. So, you just go to the nearest shop where you can buy…if you find that there are no condoms at the nearest shop then you would just risk it and go in without a condom, in the end, you even get infected” *_ Man_25–35 years.

“*Unless you have live sex without protection that is when you feel like, yes… in fact, you only use protection when a woman has insisted that you either do so or there is no sex. When we meet as men, the only sex that counts is that where no protection was used” _*Man_20–35 years.

Ultimately, individual-level factors affecting access to HIV services reflected the broader cultural and systemic factors explored in earlier sections, highlighting the interplay of stigma, gender, norms, and health system constraints.

## Discussion

Using the socio-ecological model, and enriched by social network mapping, this paper has explored factors that influence men's motivation to engage with HIV services. Our findings suggest that four overarching factors namely, stigma, pervasive social and group norms, gendered provision of HIV services and masculinity, and health systems challenges, continue to present significant challenges for men to access HIV services.

Despite progress in HIV treatment and prevention, HIV stigma was a recurring theme across all levels of the socio-ecological model. Our findings show that HIV stigma manifests differently at the individual and community levels, influencing men’s motivation to access HIV services. A 2023 qualitative study [35] from Botswana demonstrates how HIV-related gossip functions as both a manifestation and driver of stigma, permeating every level of society. This gossip driven stigma diminishes community standing and erodes psychosocial well-being, ultimately deterring individuals from accessing HIV services. This aligns with our own results, which indicate that stigma at the community and individual levels, significantly hinders men's engagement with HIV services. Together, both studies underscore the urgent need for comprehensive, culturally informed, multi-level interventions to mitigate gossip-driven stigma and normalize HIV service access. Other interventions such as virtual platforms for social support for men to interact anonymously can be explored. This approach has already been tried in another component of the study. A virtual support group for pregnant, HIV-positive women was also tried in Zambia [36]. Other evidence suggests that men are shielded from gossip and judgment when HIV services are offered in unfamiliar places or out-of-facility spaces. These places tend to offer better privacy and confidentiality for the men accessing the HIV services by encouraging the men who access the services to in turn encourage fellow men to visit these places. Evidence from Malawi and Zambia confirms that ensuring confidentiality significantly improves testing rates [29, 37].

Findings from this study show how norms, if not challenged, could negatively impact the delivery of and access to HIV services. These findings parallel those of other studies that have explored men’s access to HIV services including the influence of social and gendered norms [27, 38, 39]. Despite evidence from this community that a high percentage of men have ever tested for HIV [23], most men just assumed this was not the case because poor male engagement with HIV services has been normalized. However, the men in our study also reported that they would be willing to test for HIV if they knew other men were testing for HIV and this is in part, how descriptive norms work [32, 34]; they influence individual behavior by reflecting what is seen as normal or expected based on the actions of others in their environment. Our findings suggest that normative information could be an appropriate strategy to improve men’s uptake of HIV testing services. Normative information, which provides individuals with accurate information about descriptive norms, has been shown to increase intentions to accept a COVID-19 vaccine [40]. Promoting men’s access to credible sources of HIV information and developing and evaluating strategies that provide men with accurate information on other men’s HIV testing behaviors could motivate them to access HIV testing and related services.

An important finding of this study is how masculine traits influenced men’s access and utilization of HIV services. Men often hesitate to seek help due to fears of looking vulnerable or weak, traits that are in direct opposition to characteristics of a patient who willingly seeks health services [17, 20]. In our region and Zambia in particular, traditional views of masculinity frequently discourage both men and women from accessing healthcare, impacting their well-being [41]. This is in sharp contrast with the narrative of hegemonic masculinity, which has long portrayed men as being stronger than women and even dominating women on decisions regarding access to health services [42, 43]. However, the narrative that men fear isolation and see themselves as, and were seen as,“weak” to cope with the psychological burden of living with HIV indicate that men are not outrightly opposed to seeking care. Programs that acknowledge and address these nuanced perspectives without reinforcing stereotypes are likely to succeed in facilitating men’s access to HIV services. Our study findings also revealed some gender and age-specific dynamics. Women were key in motivating men’s access to HIV services often employing tactics like relational pressure rather than autonomous male engagement. For example, women in the 25-35 years age group used a ‘caring, family-centered approach’ by urging men to test for the sake of their family to encourage the men to prioritize their health. 

Our findings show that the health system continues to be unresponsive to men’s needs by taking a narrow rather than a holistic approach to delivering HIV services, hence the reported barriers continue to manifest as in past decades [2, 4, 17, 27, 28, 44–47]. As such, men remain a blind spot in the HIV response. In Zambia, recognizing that men are less likely to access healthcare services, men’s clinics were piloted in 2018 and have since gained traction nationally [48]. By addressing barriers related to service availability, accessibility and the feminization of the health system, these services are likely to improve men’s capability and motivation to access healthcare, including HIV, services. Whether and how these clinics improve access to HIV-testing and linkage to prevention and care services warrants exploration. In addition, the organization of the health facilities did not inspire men to access HIV services there. They saw these spaces as primarily for women and worried about being noticed in clinic queues for ART or HIV testing. This concern is more common in sub-Saharan Africa, where evidence largely indicates that men tend to engage more when services are discreet and integrated [17]. To remedy this, clinics could rethink their layouts, create private service areas, and ramp up outreach efforts especially aimed at men. They should also devise targeted strategies such as familial and social support for groups of men for which such strategies have a high chance of succeeding.

Several studies [49, 50] have highlighted the value of social support in accessing HIV services including a 2018 qualitative study working with young men in South Africa to reduce HIV risk and intimate partner violence (IPV). This study reported that men received social support to test for HIV from their female partners [16]. However, this study also revealed the tensions or dilemmas men experience when making decisions regarding whether to trust their partners and family members. The men feared abandonment and isolation including being stigmatized and gossiped if they tested HIV positive. However, this South African study also shows that support from female partners and family helps men overcome negative peer pressure and adopt healthier behaviors, like HIV testing. In our study, men who felt such support were significantly more likely to access HIV services. This convergence suggests that incorporating social support mechanisms can make a real difference in encouraging men to take charge of their health.

A strength of this study is that it takes a multi-level exploration of factors influencing men’s access to HIV services by integrating the socio-ecological model and social network mapping, offering actionable insights for targeted interventions. Social networks should be leveraged and interventions should utilize the support of peer educators and community leaders to make HIV testing and treatment feel normal. We also included views from women and stratified the groups by age to gather more nuanced information. Reshaping masculinity norms requires culturally tailored messaging that reframes health-seeking behaviour as a sign of responsibility and strength. Future studies and interventions should also look into the use of mobile testing and more decentralized testing options that reduce stigma and address logistical barriers to testing. This qualitative study included views from a total of seventy participants, 50 men and 20 women providing rich contextual data. Future research should incorporate mixed methods approaches to expand upon the insights across diverse populations.

## Conclusion

Several multi-level factors influence men’s access to HIV services. Despite men being defined as the “blind spot” in the response to HIV in 2017 [6], health systems continue to leave men behind. This study underscores the multi-level factors that negatively impact men’s motivation and capability to access HIV services. Men’s lack of social support, persistent stigma issues, and masculinity norms need to be addressed. While HIV has evolved into a chronic condition and various prevention tools are available, stigma persists in communities despite immense local and global sensitization campaigns. New interventions must enhance service delivery by prioritizing confidentiality and privacy and leveraging social networks to promote positive health behaviors. New interventions should also seek to normalize HIV testing through consistent community engagement and accurate normative information. By taking a holistic approach to addressing the various barriers and facilitators, health systems can achieve greater inclusivity and realize both regional and global goals of ending the HIV epidemic.

## Data Availability

The datasets used and/or analysed during the current study are available from the corresponding author on reasonable request..

## Abbreviations

FGD: Focus group discussion
HIV: Human immunodeficiency virus
ART: Antiretroviral Treatment
VMMC: Voluntary medical male circumcision
NHRA: National Health Research Authority (NHRA)
UNAIDS: Joint United Nations Programme on HIV/AIDS
AIDS: Acquired Immunodeficiency Syndrome
PopART: Population effects of antiretroviral therapy to reduce HIV transmission
UTT: Universal testing-and-treatment
ZAMPHIA: Zambia Population-based HIV/AIDS Impact Assessment
PrEP: Pre-Exposure Prophylaxis
PEP: Post-Exposure Prophylaxis
